# Multigene Molecular Systematics Confirm Species Status of Morphologically Convergent *Pagurus* Hermit Crabs

**DOI:** 10.1371/journal.pone.0028233

**Published:** 2011-12-09

**Authors:** Joana Matzen da Silva, Antonina dos Santos, Marina R. Cunha, Filipe O. Costa, Simon Creer, Gary R. Carvalho

**Affiliations:** 1 Molecular Ecology and Fisheries Genetics Laboratory, School of Biological Sciences, Environment Centre for Wales, Bangor University, Bangor, Wales, United Kingdom; 2 Departamento de Biologia, Centro de Estudos do Ambiente e do Mar, Universidade de Aveiro, Aveiro, Portugal; 3 Instituto Nacional de Recursos Biológicos, L-IPIMAR, Lisboa, Portugal; 4 Departamento de Biologia, Centro de Biologia Molecular e Ambiental (CBMA), Universidade do Minho, Braga, Portugal; Biodiversity Insitute of Ontario - University of Guelph, Canada

## Abstract

**Introduction:**

In spite of contemporary morphological taxonomy appraisals, apparent high morphological similarity raises uncertainty about the species status of certain *Pagurus* hermit crabs. This is exemplified between two European species, *Pagurus excavatus* (Herbst, 1791) and *Pagurus alatus* (Fabricius 1775), whose species status is still difficult to resolve using morphological criteria alone.

**Methodology/Principal Findings:**

To address such ambiguities, we used combinations of Maximum Likelihood (ML) and Bayesian Inference (BI) methods to delineate species boundaries of *P. alatus* and *P. excavatus* and formulate an intermediate *Pagurus* phylogenetic hypothesis, based upon single and concatenated mitochondrial (cytochrome oxidase I [COI]) and nuclear (16S and 28s ribosomal RNA) gene partitions. The molecular data supported the species status of *P. excavatus* and *P. alatus* and also clearly resolved two divergent clades within hermit crabs from the Northeast Atlantic Ocean and the Mediterranean Sea.

**Conclusions/Significance:**

Despite the abundance and prominent ecological role of hermit crabs, *Pagurus*, in North East Atlantic Ocean and Mediterranean Sea ecosystems, many important aspects of their taxonomy, biology, systematics and evolution remain poorly explored. The topologies presented here should be regarded as hypotheses that can be incorporated into the robust and integrated understanding of the systematic relationships within and between species of the genus *Pagurus* inhabiting the Northeast Atlantic Ocean and the Mediterranean Sea.

## Introduction

Although hermit crabs are one of the most morphologically and ecologically diverse group of decapod crustaceans, their evolutionary history at many taxonomic levels is far from being resolved [1]–[4]. More than 1000 species, 127 genera and 6 families are currently reported for the superfamily Paguroidea [5], that inhabit diverse biotopes from intertidal to deep seas [6]. However, the true taxonomic and ecological heterogeneity associated with hermit crabs is likely to be underestimated because many species and life-histories appear to be undescribed [4]. One of the most diverse groups belong to the family Paguridae, with species distributed widely through all oceans [5].

The genus *Pagurus* Fabricius, 1775 is considered to be ancient, with fossils being assigned to the genus as early as the lower Cretaceous [7] and from the Cenozoic [8]. *Pagurus* is the least complex of all paguroids, sharing a reduced or virtually nonexistent rostrum, no sexually modified appendages other than the regular female egg-bearing pleopods, and having no penis (or sexual tubes) [3]. Despite its comparatively morphological conservatism, *Pagurus* exhibits a high degree of species proliferation. Recently, 172 species (of which five are fossil records) are recognised [9] , that possess specialized adaptations for housing stability, relying upon gastropod shells for protection. Such commensalism has constrained morphological evolution over 150 million years [7], [8] by requiring a decalcified asymmetrical abdomen capable of looping into gastropod shells. Despite the abundance of hermit crabs in the North East Atlantic and Mediterranean Sea [6] many important aspects of their taxonomy [10], biology [11]–[18], ecology [17], [19]–[22], systematics and evolution [3], [8], [23] are poorly documented.

Systematic problems remain among the Paguridae, such as the polyphyletic genus *Pagurus* Fabricius, 1775 [4]. To date, most systematic studies on hermit crabs have been based on morphology with relatively few studies utilising molecular tools to resolve species status [4], [24]–[26] or to determine phylogenetic relationships among major taxa [2], [4], [23]. Within the region of the Northeast Atlantic Ocean and Mediterranean Sea *Pagurus* is represented by 23 species [6] and high morphological similarity among some species has resulted in the recognition of two groups characteristic of the North Atlantic and Mediterranean Sea [10]. In some cases, morphology suggests very close relationships between congeners, raising uncertainty about their independent species status. Such a situation exists between the “alatus” group, *Pagurus excavatus* (Herbst, 1791) and *Pagurus alatus* (Fabricius, 1775) that is still difficult to resolve using morphology alone (Table 1) [6], [10]. In spite of the prevailing taxonomic challenges, Ingle [10] has recognized both species, based mainly from the differences observed in the dorsal aspects of shield, antennular peduncle, the shape of the right cheliped, the length of larger pereiopod and male pleopods. Furthermore, due to a lack of life history studies there has always been considerable confusion regarding ongoing synonymies that are assigned among *P. alatus*, *P. excavatus* and *P. variabilis* (A. Milne-Edwards & Bouvier, 1892) [10], [27]. As currently recognised, *P. excavatus* is distributed southwards from the southern part of the Bay of Biscay and into the Mediterranean Sea. *Pagurus alatus* extends northwards into Iceland waters, but remains sympatric with *P. excavatus* in many southern regions of the area [10]. Here, we use molecular phylogenetic analyses of mitochondrial cytochrome oxidase I (COI), mitochondrial 16S ribosomal RNA (16S), and nuclear 28S ribosomal RNA (28S) DNA partitions to reconstruct the systematics of selected *Pagurus* species, in order to make inferences on taxonomic status of *P. alastus* and *P. excavatus.*

**Table 1:** Selected morphological characters by Ingle (1985) to distinct *Pagurus alatus* (Fabricius 1775) vs *Pagurus excavatus* (Herbst 1791).

Morphological selected characters by Ingle (1985)	
Dorsal aspect of shield and associated appendages	
*Pagurus alatus* (Fabricius 1775)	*Pagurus excavatus* (Herbst 1791)
Segment 1 of **antennular** peduncle, outer **distal margin with one, two or sometimes three spines**.	Segment 1 of **antennular** peduncle, outer **distal margin without or with small obtuse spines at the most**.
Outer dorso-lateral process of **antenna** (of large circa 80 mm SL, specimens) **reaching just beyond distal margin** of segment 4 and **acicle** reaching **well beyond distal extremity of cornea**; breadth of **cornea** slightly **exceeding 1\2 length of eye**.	Outer dorso-lateral process of **antenna** (of large specimens circa 80 mm SL) **not reaching to distal margin** of segment 4 and **acicle** reaching **only to extremity of cornea**; breadth of **cornea** slightly **less than 1\2 length of eye**.
Outer (particularly upper) surface of right **cheliped palm not strikingly concave**.	Outer upper (and sometimes lower) surface of right **cheliped palm strikingly concave**.
**Larger pereiopod 3**, **dactyl** (of large specimens, circa 10 mm SL) **as long as combined lengths** of propodus + carpus and noticeably curved; **males** with **3 unpaired pleopods**.	**Larger pereiopod 3**, **dactyl** (of large specimens, circa 9–10 mm SL) **slightly longer than combined** lengths of propodus + carpus and noticeably curved; **males** with **4 unpaired pleopods**.

Words in bold are highlighting the solely differences between species and “SL” is the abbreviation of shield length.

## Results

### *Pagurus* diversity

**Figure 1:**
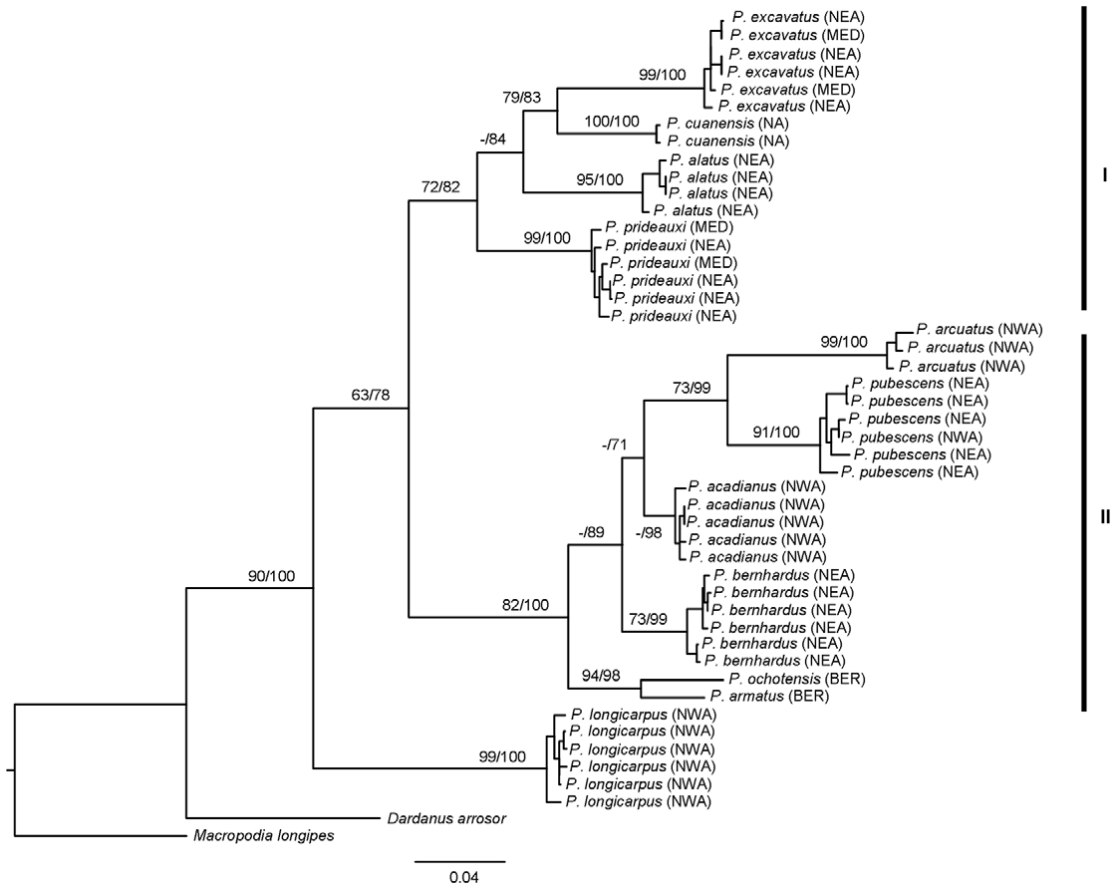
BI phylogram of the 46 COI sequences of 11 *Pagurus* species selected. The numbers on branches are ML bootsrap values and posterior probabilities of BI<50% (ML/BI percentages values, respectively). Each oceanographic region/specimens are defined: Northeast Atlantic Ocean (NEA), Northwest Atlantic Ocean (NWA), North Atlantic Ocean (NA), Mediterranean Sea (MED) and Bering Sea (BER) (see Table 4 for complement information).Two major clades have been roman number-coded, I and II: represent two groups defined previously by Ingel (1985) based on adult and larvae morphological characters.

The variation in COI diversity was examined among 11 species (Table 2 and 3). For each species, one to six representative individuals were analysed (Table 4), and where possible, from different geographical areas, yielding a total of 46 sequences. No insertions, deletions, stop codons or sequences indicative of pseudogenes [28], [29] were observed, and BLAST searches confirmed that the sequences corresponded to decapod mtDNA COI. There was also evidence for base composition bias in the sequences, notably a pronounced underrepresentation of guanine at the third codon positions (35.3% T; 18.1% C; 28.5% A; 18.1%G) a phenomenon commonly observed in metazoan mitochondrial [30]. The COI alignment contained a total of 513 bp with 177 variable characters, of which 170 were parsimony informative (33.13%). The high observed percentage of parsimony-informative character suggests that COI is sufficiently diverse for intrageneric phylogeny and clearly resolved all eleven *Pagurus* species examined in the present study (Figure 1) [31], [32]. The 11 species comprise a monophyletic clade (Figure 1) with an average between genetic species distance of 17.01% (Table 2). Among the *Pagurus* species, *P. acadianus* exhibits the least genetic divergence to *P. bernhardus* (6.80%) and in contrast, *P. arcuatus* and *P. excavatus* exhibited the highest genetic distances (23.10%) (Table 3). *P. pubescens* exhibited the highest average distance values (Table 3) with a range of 11.50–21.70% (see also Figure 1).

**Table 2:** Pairwise COI nucleotide divergences for *Pagurus* spp using K2P distances (%).

Pairwise divergences comparisons	n	Taxa	Min Dist(%)	Mean Dist(%)	Max Dist(%)	SE Dist(%)
***Pagurus*** ** (11 species)**						
Within a species*	44	9	0	0.632	2.002	0.055
Between species	46	1	6.432	17.019	23.086	0.106

*Number of specimens with more than 1 sequences analysed.

**Table 3:** Pairwise COI nucleotide divergences for each selected *Pagurus* spp using K2P distances (%).

Species	Distances (%)											
	Within species	Between species										
		1	2	3	4	5	6	7	8	9	10	11
1 *Pagurus acadianus* (Benedict, 1910)	0.2											
2 *Pagurus alatus* (Fabricius, 1775)	0.5	17.70										
3 *Pagurus arcuatus* (Squires, 1964)	1.2	13.80	17.50									
4 *Pagurus bernhardus* (Linnaeus, 1758)	0.7	6.80	16.70	14.00								
5 *Pagurus cuanensis* (Bell, 1845)	0	17.80	13.40	19.50	18.00							
6 *Pagurus excavatus* (Herbst, 1791)	0.1	19.50	14.80	23.10	20.00	13.00						
7 *Pagurus prideauxi* (Leach, 1815)	0.4	18.20	13.70	20.50	17.30	15.00	15.60					
8 *Pagurus pubescens* (Krøyer, 1838)	1.6	11.50	17.70	13.90	12.70	18.90	21.70	21.20				
9 *Pagurus longicarpus* (Say, 1817)	0.6	19.30	16.20	19.80	18.80	20.50	21.30	18.70	19.80			
10 *Pagurus armatus* (Dana, 1851)	-	12.20	21.40	19.80	12.90	21.10	22.80	21.30	17.80	21.70		
11 *Pagurus orchotensis* (Brandt, 1851)	-	13.50	20.00	16.90	14.90	20.90	21.20	21.50	19.30	21.10	9,20	

**Table 4:** *Pagurus* and outgroup specimens used for the COI phylogenetic reconstructions.

Species	Collection site	GenBank
		accession No.
*Pagurus acadianus* (Benedict, 1901)	Maine (United States)	AF483156
*Pagurus acadianus* (Benedict, 1901)	Prince Edward Island (Canada)	FJ581812
*Pagurus acadianus* (Benedict, 1901)	Quebec (Canada)	FJ581815
*Pagurus acadianus* (Benedict, 1901)	Quebec (Canada)	FJ581814
*Pagurus acadianus* (Benedict, 1901)	Quebec (Canada)	FJ581813
*Pagurus alatus* (Fabricius, 1775)	Costa Algarvia (Portugal)	JN107574
*Pagurus alatus* (Fabricius, 1775)	Costa Algarvia (Portugal)	JN107575
*Pagurus alatus* (Fabricius, 1775)	Costa Algarvia (Portugal)	JN107576
*Pagurus alatus* (Fabricius, 1775)	Costa Algarvia (Portugal)	JN107577
*Pagurus arcuatus* (Squires, 1964)	Quebec (Canada)	FJ581818
*Pagurus arcuatus* (Squires, 1964)	Quebec (Canada)	FJ581817
*Pagurus arcuatus* (Squires, 1964)	Quebec (Canada)	FJ581816
*Pagurus armatus* (Dana, 1851)	Nova Scotia (Canada)	AF483159
*Pagurus bernhardus* (Linnaeus, 1758)	Wales (United Kingdom)	JN107580
*Pagurus bernhardus* (Linnaeus, 1758)	Wales (United Kingdom)	JN107581
*Pagurus bernhardus* (Linnaeus, 1758)	Wales (United Kingdom)	JN107582
*Pagurus bernhardus* (Linnaeus, 1758)	Costa de Prata (Portugal)	JN107583
*Pagurus bernhardus* (Linnaeus, 1758)	England (United Kingdom)	JN107578
*Pagurus bernhardus* (Linnaeus, 1758)	England (United Kingdom)	JN107579
*Pagurus cuanensis* (Bell, 1845)	Azores (Portugal)	JN107584
*Pagurus cuanensis* (Bell, 1845)	Azores (Portugal)	JN107585
*Pagurus excavatus* (Herbst, 1791)	Costa de Prata (Portugal)	JN107586
*Pagurus excavatus* (Herbst, 1791)	Costa de Prata (Portugal)	JN107587
*Pagurus excavatus* (Herbst, 1791)	Costa de Prata (Portugal)	JN107590
*Pagurus excavatus* (Herbst, 1791)	Costa de Prata (Portugal)	JN107591
*Pagurus excavatus* (Herbst, 1791)	Sicily (Italy)	JN107588
*Pagurus excavatus* (Herbst, 1791)	Sicily (Italy)	JN107589
*Pagurus longicarpus* (Say, 1817)	New Brunswick (Canada)	FJ581825
*Pagurus longicarpus* (Say, 1817)	New Brunswick (Canada)	FJ581824
*Pagurus longicarpus* (Say, 1817)	New Brunswick (Canada)	FJ581823
*Pagurus longicarpus* (Say, 1817)	New Brunswick (Canada)	FJ581822
*Pagurus longicarpus* (Say, 1817)	New Brunswick (Canada)	FJ581826
*Pagurus longicarpus* (Say, 1817)	Nova Scotia (Canada)	FJ581820
*Pagurus ochotensis* (Brandt, 1851)	Alaska (United States)	AF483158
*Pagurus prideauxi* (Leach, 1815)	England (United Kingdom)	JN107597
*Pagurus prideauxi* (Leach, 1815)	Costa de Prata (Portugal)	JN107595
*Pagurus prideauxi* (Leach, 1815)	Costa de Prata (Portugal)	JN107596
*Pagurus prideauxi* (Leach, 1815)	Sicily (Italy)	JN107592
*Pagurus prideauxi* (Leach, 1815)	Sicily (Italy)	JN107593
*Pagurus prideauxi* (Leach, 1815)	Bear Island Slide (Norway)	JN107594
*Pagurus pubescens* (Krøyer, 1838)	Quebec (Canada)	FJ581829
*Pagurus pubescens* (Krøyer, 1838)	Bear Island Slide (Norway)	JN107598
*Pagurus pubescens* (Krøyer, 1838)	Bear Island Slide (Norway)	JN107599
*Pagurus pubescens* (Krøyer, 1838)	Svalbard (Norway)	JN107600
*Pagurus pubescens* (Krøyer, 1838)	Svalbard (Norway)	JN107601
*Pagurus pubescens* (Krøyer, 1838)	Svalbard (Norway)	JN107602
*Dardanus arrosor* (Herbst, 1796)	Sicily (Italy)	JN107572
*Macropodia longipes* (Milne-Edwards & Bouvier, 1899)	Costa de Prata (Portugal)	JN107573

### Phylogenetic relationships

Overall, the phylogenetic algorithms (ML, BI) resulted in congruent topologies, delimiting the designated true *Pagurus* species (Figure 1). The resulting molecular phylogeny agrees in several respects with the current morphologically based classification of all species. All analyses support the basal placement of *P. longicarpus* and identify two main clades (Figure 1). In contrast, the relationships among inner clades of *Pagurus* were poorly resolved. Clade I is represented by four the Northeast Atlantic Ocean and Mediteranean Sea species and clade II by six species of the North Atlantic Ocean (East and West coasts) and Bering Sea specimens.

### Molecular systematic assignments

The three independent genes revealed concordant phylogenetic differences between *P. alatus* and *P.excavatus*. *Pagurus alatus* is substantially divergent from *P. excavatus*, with a mean divergence of 14.9% and 5.1% for COI and 16S sequences respectively (Table 5). The 16S alignment was more conserved than the COI partition yielding 84/462 variable characters, of which 75/462 were parsimony informative. The 16S sequences are AT-rich (71.86%), indicating a moderate compositional bias. The pattern of nucleotide substitution was also biased in favour of transitions over transversions, yielding a ts∶tv = 1.1 and for 28S a ts∶tv = 3.2.

**Table 5:** Sequence identity matrix estimated from 16S with TVM+G model (above diagonal) and COI (below diagonal) with TIM2+I+G model between selected *Pagurus* species of Northeast Atlantic Ocean and Mediterranean Sea.

Species	Collection site	1	2	3	4	5	6	7	8	9	10	11	12	13	14
1 *Pagurus alatus* (F*abricius, 1775)*	Costa Algarvia (Portugal)		0.02	0.02	0.04	1.81	0.61	0.57	0.57	0.5	0.53	0.5	0.5	1.92	1.83
2 *Pagurus alatus* (F*abricius, 1775)*	Costa Algarvia (Portugal)	0.1		0	0.02	1.77	0.59	0.55	0.55	0.47	0.5	0.47	0.47	1.87	1.79
3 *Pagurus alatus* (F*abricius, 1775)*	Costa Algarvia (Portugal)	0	0.1		0.02	1.77	0.59	0.55	0.55	0.47	0.5	0.47	0.47	1.87	1.79
4 *Pagurus alatus* (F*abricius, 1775)*	Costa Algarvia (Portugal)	0	0.1	0		1.81	0.61	0.57	0.57	0.5	0.53	0.5	0.5	1.83	1.83
5 *Pagurus bernhardus* (Linnaeus, 1758)	Wales (United Kingdam)	5.35	4.69	5.35	5.35		1.81	1.74	1.74	1.9	1.89	1.9	1.9	0.56	0.51
6 *Pagurus excavatus* (Herbst, 1791)	Sicily (Italy)	3.82	3.59	3.82	3.82	7.04		0.02	0.02	0.8	0.77	0.8	0.8	2.05	1.97
7 *Pagurus excavatus* (Herbst, 1791)	Sicily (Italy)	3.82	3.59	3.82	3.82	7.04	0		0	0.76	0.73	0.76	0.76	1.98	1.9
8 *Pagurus excavatus* (Herbst, 1791)	Costa de Prata (Portugal)	3.82	3.59	3.82	3.82	7.04	0	0		0.76	0.73	0.76	0.76	1.98	1.9
9 *Pagurus prideauxi* (Leach, 1815)	Bear Island Slide (Norway)	3.56	3.39	3.56	3.56	4.99	4.05	4.05	4.05		0.02	0	0	2.01	1.93
10 *Pagurus prideauxi* (Leach, 1815)	Wales (United Kingdam)	3.65	3.48	3.65	3.65	5.1	4.15	4.15	4.15	0.02		0.02	0.02	2	1.92
11 *Pagurus prideauxi* (Leach, 1815)	Costa Algarvia (Portugal)	3.65	3.48	3.65	3.65	5.03	4.15	4.15	4.15	0.04	0.02		0	2.01	1.93
12 *Pagurus prideauxi* (Leach, 1815)	Sicily (Italy)	3.65	3.48	3.65	3.65	5.1	4.15	4.15	4.15	0.02	0	0.02		2.01	1.93
13 *Pagurus pubenscens* (Krøyer, 1838)	Svalbard (Norway)	5.33	5.02	5.33	5.33	2.7	7.12	7.12	7.12	7.29	7.44	7.44	7.44		0.04
14 *Pagurus pubenscens* (Krøyer, 1838)	Svalbard (Norway)	5.64	5.26	5.64	5.64	2.85	7.07	7.07	7.07	7.17	7.32	7.32	7.32	0.18	

All values are expressed as percentage.

**Figure 2:**
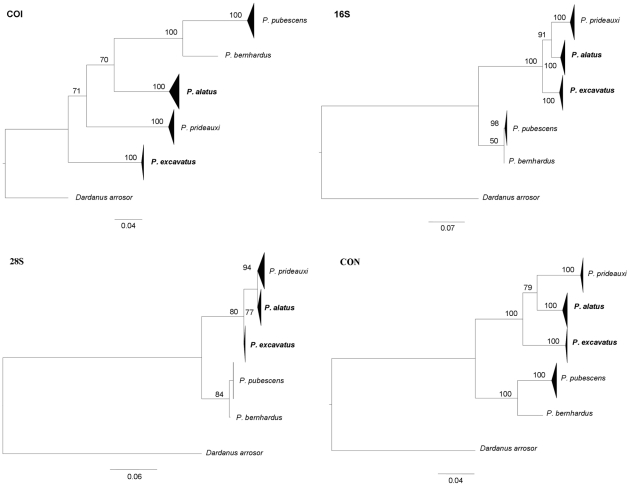
BI phylograms of each individual gene (COI, 16S, 28S) and concatenated data set (CON = COI+16S+28S). The numbers on branches are posterior probabilities >50% of BI (in percentage). Species *P. alatus* and *P.excavatus* are highlighter a bold.

In the BI analysis, systematic positions of five species were not stable (Figure 2 A), but the trees underpinned by the 16S, 28S and the concatenated data partitions were broadly congruent with the COI hypothesis (Figure 1). The combined molecular analysis, based on strong posterior probabilities values (100%; in Figure 2, CON), clearly reveals the two independent lineages here discussed.

## Discussion

### *Pagurus* diversity

Systematics work to date in *Pagurus* has primarily been in the area of morphological taxonomy, with few hypotheses presented regarding species relationships. Generating such inferences has been difficult because of generally remarkable similarities in morphology among members of the genus. These similarities are perhaps surprising given the degree of the genetic divergence observed in this study between the most phenotypically closest species, *P. alatus* and *P. excavatus* (14.8%). A similar pattern has been observed in penaied shrimps [33], porcelanids crabs [34], diogenid hermit crabs [35] and has been attributed to stabilizing selection on morphological/ecological characters, or that they are on independent evolutionary trajectories. Likewise, strong stabilizing selection acting on morphological differentiation has been associated with life as a commensal of thallassinidean shrimps, accompanied by neutral molecular divergence [36].

The smallest mean intraspecific divergence values observed (Table 3) are possibly underestimated, because samples were obtained from a single locality. Global-scale phylogeography surveys of COI sequence diversity have estimated average intraspecific diversity values of less than 1% within crustaceans, whereas interspecific values typically are greater than 4% among congeneric species [37]–[39] and especially among decapods that can exhibit congeneric divergence values greater than 15% [40]. Elsewhere, five species of the genus *Pagurus* from Sea of Japan exhibited lower levels of genetic identity when compared with the genera *Metapenaeus* and *Penaeus* [25]. Here, the high genetic diversity observed among the pagurid species is in line with the observed morphological variability in informal morphological groups among adults and larvae described by Ingle (1985) and McLaughlin and Gore (1988), respectively.

### Phylogenetic relationships

Ingle [10] delineated two main groups of Northeast Atlantic Ocean and Mediterranean Sea *Pagurus* based on three adults, and additional larval morphological characters [10] that are fully congruent with the current molecular systematic analysis. Furthermore, *P. armatus*, *P. ochotensis* and *P. bernhardus* that were assumed to be most related, based on morphology [10], share the most basal position in clade II. In addition, all species from clade I and II agree with two distinct larvae groups described by McLaughlin and Gore [41] based on the characteristics and species assigned. The inconsistent phylogenetic position of *P. bernhardus*, observed in Mantelatto *et al.* [4] was still unresolved here (Figure 1), represented by the weak support of the two focal nodes (<50% bootstrap support) within clade II. However, the observed COI molecular divergence does support a sister group relationship between *P. acadianus* and *P. bernhardus*, a taxonomic relationship derived also from morphological comparisons [7], [42]. In summary, the phylogenetic patterns observed for *Pagurus* are consistent with morphological groups established by Ingle [10] and McLaughlin and Gore [41] but further analyses would be required to establish the precise cladistic relationships within the genus as a whole.

### Molecular systematic assignments

Concordance across molecular and morphological characters provides a reliable indicator of longstanding evolutionary independence and consequently provides an operational criterion for species recognition [43]. In the present study, morphological differences between *P. alatus* and *P. excavatus* were evident when two morphological variants were compared directly (Table 1), supporting molecular findings that justify separation at the species level. It is important to mention that a restricted collection from one biogeographical region of a species with a wide distribution cannot represent the complete range of morphological variation that is often found in decapods [4], [27], [44]. We also know that phenotypic plasticity in morphological traits (especially organisms with commensal behaviours) might be strongly influenced by environmental factors between different areas [45]–[47]. The precise cladistic relationships among the five species were not stable (Figure 2, A), most likely due to lower taxon sampling and also the inability of COI to accurately resolve deep nodes. However, the combined molecular analysis (Figure 2, CON) clearly reveals the evidence for long-standing evolutionary independence between *P. excavatus* and *P. alatus*.

### Conclusion

Molecular data have not been used before to investigate systematic relationships among *Pagurus* of the Northeast Atlantic Ocean and Mediterranean Sea. Despite the perceived limitations regarding the use of morphological characters for inferring evolutionary relationships among commensal species, our molecular data support the morphological taxonomy. Our data may indicate the possible existence of two monophyletic groups (clade I and II), supporting previous assertions based on larval and adult morphological criteria. However, the current data confirm the complexity of the relationships within *Pagurus*, highlighting the absence of complete and integrated morphological descriptions for the diverse and heterogeneous members of the genus. Since the present taxonomic and geographic coverage is incomplete, the topologies presented here should be regarded as working hypotheses. *Pagurus* have diversified into a wide variety of marine habitats and exemplify classic commensal, anti-predator evolutionary traits. Thus, the group provides an excellent model for studying the interplay between speciation, neutral molecular divergence and potential stabilising selection on body form.

## Materials and Methods

### Sampling

Twenty nine hermit crabs of six species, *Pagurus alatus* (Fabricius, 1775), *P. bernhardus* (Linnaeus, 1758), *P. cuanensis* (Bell, 1845), *P. excavatus* (Herbest, 1791), *P. prideaux* (Leach, 1815), and *P. pubescens* (Krøyer, 1838), were collected from the Portugal (Costa Algarvia, Costa de Prata, and Azores), United Kingdom (North Wales), Norway (Bear Island Slide and Svalbard) and Italy (Sicily) between 2006 and 2008. Specimens were harvested as by-catch from rough ground bottom trawls from INRB-IPIMAR, Sicilian fisheries survey and collected by physical searches. Species were identified (Ingle, 1985) prior to the excision and preservation of muscle tissue in 95% ethanol (stored at −20°C) and whole body storage at −20°C. In order to accurately assign specimens to currently accepted (female) species of *P. alatus* and *P. excavatus* we used morphological criteria based upon four main groups of characters: the dorsal aspects of shield (Table 1), antennular peduncle, the shape of the right cheliped, length of larger pereiopod and male pleopods (Ingle 1985).

### DNA isolation, amplification and sequencing

Total genomic DNA was extracted from approximately 1 mm^3^ of muscle tissue or whole legs for small specimens using the Chelex dry release method [48]. The COI gene was amplified with alternative sets of primers depending on PCR reaction success following by the protocol develop by Costa *et al*. [34]. All PCRs were performed in a 25 µl final volume containing 1× PCR buffer, 3–4 mM MgCl_2_, 0.1–0.3 mM dNTP, 1 U *Taq* polymerase, 5–10 pmol of each primer, and 2–10 ng of DNA template (Table 6). The thermal cycling conditions are listed in Table 6 for each set of primers. Following amplification, PCR reactions were cleaned by incubation with 10 U Exonuclease I (New England Biolabs ®) and 1 U Shrimp Alkaline Phosphate (Promega ®) at 37°C for 1 hour, followed by heating at 80°C for 5 min. Samples were sequenced by Macrogen Inc. (South Korea) using an Applied Biosystems® 3730 sequencer.

**Table 6:** Primers sequences and thermocycling conditions for the amplification reactions.

Locus and			
primers names	Sequences	Reference	Cycling conditions
**COI**			
Forward			
LCOI490	5′-GGTCAACAAATCATAAAGATATTGG-3′	Folmer *et al.*, [68]	Denaturation 94°C/60 s;
CrustF1	5′-TTTTCTACAAATCATAAAGACATTGG-3′	Costa *et al.*, [37]	35–40 cycles at 94°C/30 s,
Reverse			48–56°C/90 s, 72°C/60 s;
HCO2198	5′-TAACTTCAGGGTGACCAAAAAATCA-3′	Folmer *et al.*, [68]	Final extension at 72°C/5 min.
			Denaturation 94°C/60 s;
			5 cycles at 94°C/30 s,
			45°C/90 s, 72°C/60 s;
			35–40 cycles at 94°C/30 s,
			50–56°C/90 s, 72°C/60 s;
			Final extension at 72°C/5 min.
**16S**			
Forward			
16SL2	5′-TGCCTGTTTATCAAAAACAT-3′	Mathews *et al.*, [69]	Denaturation 94°C/3 min.;
16Sar	5′-CGCCTGTTTATCAAAAACAT-3′	Palumbi *et al*., [33]	35–40 cycles at 94°C/30 s,
Reverse			50–55°C/60 s, 72°C/2 min.;
16S-1472	5′-AGATAGAAACCAACCTGG-3′	Schubart *et al.*, [70]	Final extension at 72°C/5 min.
16SBr-Dr	5′-CCGGTTTGAACTCAGATCATG-3′	Palumbi *et al.*, [33]	
**28S**			
Forward			
28S-rD1.2a	5′- CCCSSGTAATTTAAGCATATTA-3′	Whiting *et al*., [71]	Denaturation 94°C/5 min.;
Reverse			35 cycles at 95°C/30 s,
28SRd3.2b1	5′-TYAACGGTTTCACGTRCTMTTGA-3′	This study	50°C/45 s, 72°C/60 s;
			1 cycle at 95°C/30 s,
			45°C/45 s and final extension
			at 72°C/5 min.

All empirically derived sequences were manually checked for ambiguities in CodonCode Aligner version 1.3.0, aligned using the ClustalX plugin embedded within Mega 4 [49], prior to manual quality control and megablast annotation.

### Phylogenetic relationships

The most popular barcode marker COI is generally used to study close to moderately deep interspecific taxon relationships of crustaceans [50]–[54]. To provide a comprehensive sister-species coverage and assessment of interspecific variation, *Pagurus* COI sequences from GenBank were merged with our data (Table 4). In that propose we use using Kimura 2-parameters (K2P) genetic distances within and among species implemented in Mega 4.1, and compared to literature data. Amino acid translations of the target genes were examined to ensure that no gaps or stop codons were present in the alignment.

To identify phylogenetic groups among the resulting eleven putative *Pagurus* species, the 46 COI sequences comprising 513 bp was analyzed using Maximum Likelihood (ML) and Bayesian Inference (BI) phylogenetic reconstruction methods. The crab *Macropodia longipes* (Milne-Edwards & Bouvier, 1899) (Brachyura:Inachidae) and the most phylogenetically closest related species, *Dardanus arrosor* (Herbst, 1796) (Anomura: Diogenidae) were used as outgroups.

Since Bayesian posterior probability support values (bpp) can often be inflated for certain clades, relative to ML bootstrap values [55], we constructed trees using both Bayesian approaches and ML. For these searches, we set the substitution model parameters calculated by jModeltest in RAxML 7.0.4 [56] and in MrBayes 3.1 [57], respectively. Ten independent ML analyses were conducted using GTR+I+G with invariant sites (I) and gamma distributed rates (G) [58] (see below) as the model (using the GTR+CAT setting) with 4 categories of rate variation (500 bootstrap replicates were undertaken for estimation of node support) for each partition on combined data. In order to find the ML tree, 10 independent runs of RAxML 7.0.4 were conducted. ModelTest [59] identified the HKY+I+G model [60] as best indicated by Akaike Information Criterion (AIC) [61], however, since this model is not implemented in the current version of RAxML, the GTR+I+G model was selected as the closest matching alternative. In MrBayes, two independent Markov chain Monte Carlo (MCMC) analyses were run using four chains for 5×10^6^ generations with the initial 1 million generations (20%) cycles discarded as burn-in. To check that stationarity had been reached, we monitored the fluctuating value of the likelihood graphically with Tracer v1.4 [62]. Once the parameters reach stationarity, a 50% majority rule consensus tree was obtained from the remaining saved trees. The consensus tree was selected from the posterior distribution and visualized using FigTree V.1.0 (http://tree.bio.ed.ac.uk/software/figtree/). Since substitution rates among the four nucleotides and among different nucleotide sites in mitochondrial protein-coding genes as been reported [63]–[65], codon models of substitution TrNef+G [66], F81 [67] and HKY+G [60] were implemented in our BI analyses for first, second and third codon positions respectively.

### Molecular systematic analyses

Use of nuclear genes in addition to mitochondrial genes adds to the number of independent markers in a dataset, thus increasing the chances to understand the systematic relationships between and within *P. alatus* and *P. excavatus* (Table 7). Here we analysed partial sequences of nuclear 28S (385 bp), mitochondrial 16S (462 bp), and the barcode region of COI (540 bp). The three gene regions were partitioned separately according to the previously determined model parameters (Table 8) in subsequent BI analyses. Gaps in 16S and 28S sequences were treated as a fifth character-state. BI analysis was conducted for each gene data set and the concatenated partition (CON) with the three gene regions partitioned separately according to the previously determined model parameters (Table 8) as described before. To evaluate the range of intrageneric sequence identity found among *Pagurus* species, we compared pairwise distances of COI and 16S (Table 5).

**Table 7:** Selected Northeast Atlantic Ocean and Mediterranean Sea *Pagurus* species and outgroup *Dardanus* species for molecular systematic reconstructions with respective date and site of collection, museum catalogue number, and genetic database accession numbers (Genbank).

Species	Collection site	Catalogue	Genbank accession No.		
		No.	COI	16S	28S
*Pagurus alatus* (Fabricius, 1775)	Costa Algarvia (Portugal)	MB89000415	JN107574	JN107604	JN107621
*Pagurus alatus* (Fabricius, 1775)	Costa Algarvia (Portugal)	MB89000418	JN107575	JN107606	JN107622
*Pagurus alatus* (Fabricius, 1775)	Costa Algarvia (Portugal)	MB89000450	JN107576	JN107607	JN107619
*Pagurus alatus* (Fabricius, 1775)	Costa Algarvia (Portugal)	MB89000463	JN107577	JN107605	JN107620
*Pagurus bernhardus* (Linnaeus, 1758)	Wales (United Kingdom)	MB89000491	JN107579	JN107608	JN107623
*Pagurus excavatus* (Herbst, 1791)	Costa de Prata (Portugal)	MB89000268	JN107586	JN107609	JN107626
*Pagurus excavatus* (Herbst, 1791)	Sicily (Italy)	MB89000078	JN107588	JN107610	JN107627
*Pagurus excavatus* (Herbst, 1791)	Sicily (Italy)	MB89000079	JN107589	JN107611	JN107628
*Pagurus prideauxi* (Leach, 1815)	Costa de Prata (Portugal)	MB89000311	JN107596	JN107614	JN107631
*Pagurus prideauxi* (Leach, 1815)	Sicily (Italy)	MB89000086	JN107593	JN107615	JN107632
*Pagurus prideauxi* (Leach, 1815)	Wales (United Kingdom)	MB89000492	JN107597	JN107613	JN107630
*Pagurus prideauxi* (Leach, 1815)	Bear Island Slide (Norway)	MB89000493	JN107594	JN107612	JN107629
*Pagurus pubenscens* (Krøyer, 1838)	Svalbard (Norway)	MB89000489	JN107601	JN107616	JN107633
*Pagurus pubenscens* (Krøyer, 1838)	Svalbard (Norway)	MB89000490	JN107602	JN107617	JN107633
*Dardanus arrosor* (Herbst, 1796)	Sicily (Italy)	MB89000494	JN107572	JN107603	JN107618

**Table 8:** Substitution models for the molecular systematic analyses of selected *Pagurus* species from Northeast Atlantic Ocean and Mediterranean Sea.

Gene	Substitution model	Among-site rate variation*		Base frequencies			
		I	Γ	A	C	G	T
COI**	TIM2	0.454	0.41	0.3104	0.1484	0.1625	0.3787
1st codon	TrN	0.629	-	0.2901	0.1667	0.307	0.2363
2nd codon	F81	-	-	0.1306	0.2565	0.1688	0.4441
3rd codon	TPM3uf	-	0.622	0.4405	0.0937	0.0724	0.3935
16S	TVM	-	0.382	0.3532	0.1048	0.1766	0.3654
28S	TrN	0.327	0.258	0.1889	0.2976	0.2282	0.2852

*I proportion of invariable sites; Γ, gamma distribution shape parameter. I and Γ values refer to the AIC.

**COI partition represented by first (1^st^), second (2^nd^) and third (3^rd^) codon positions.
